# Supplementary material for: The role of genetic diversity and arbuscular mycorrhizal fungal diversity in population recovery of the semi-natural grassland plant species Succisa pratensis
Source: BMC Ecol Evol. 2021 Nov 5;21:200. doi: 10.1186/s12862-021-01928-0 (PMC8570031; doi:10.1186/s12862-021-01928-0)
Supplement: Supplementary file 1 — Additional file 1: Table S1. Twelve loci were amplified to assess the intraspecific genetic variation of Succisa pratensis using Single Sequence Repeats (SSR). [file 12862_2021_1928_MOESM1_ESM.docx]

**Table S1** Twelve loci were amplified to assess the intraspecific genetic variation of *Succisa pratensis* using Single Sequence Repeats (SSR).

| **Locus** | **Forward primer** | **Reverse primer** | **Motif** | **Alleles** | **Base pairs** |
| --- | --- | --- | --- | --- | --- |
| Supr_10 | TTTGTTCGCATTTCTCTTCC | GCCGCGATGAAGATAAAGAA | AAG | 6 | 94-109 |
| Supr_12 | TGATCAAAGTTGAACAACGAGAA | AAACCCAAAGCATACTTGTGAAA | AC | 5 | 116-124 |
| Supr_13 | CATCAGTGGTCTACGTTTGTGA | TGGATTTAGTAAGTGACTGTGCTTT | TA | 8 | 133-151 |
| Supr_14 | CTCTCAGCCTTTTGGTCTGG | TCAGTGTGGAAAAGCTAGTTCG | AT | 5 | 178-188 |
| Supr_23 | CCGAGGAAGATGGTACTGGA | CGTAACATGCCACAACAACC | GGA | 3 | 106-115 |
| Supr_30 | CCACAAATACAAGAACATCACCA | TCCTCTTTTGAAGCTTTCGC | AT | 2 | 206-208 |
| Supr_31 | GGGAGGTTGCCAAATGACTA | TTTGACGGGTAGTTGAAGGG | AT | 5 | 256-268 |
| Supr_32 | CACCTGACGCATGTGAACTT | GATCCTCGCGATGACTCACT | AT | 3 | 311-315 |
| Supr_34 | TCAAGCAACCAAGACTAGGGA | GAAGCAACAGAATGGAGATTTG | TCT | 4 | 108-120 |
| Supr_35 | CACCCTTAAATTTCATAAATGCAC | TTTTGTTTGTGCTAAATGGGG | TA | 4 | 79-86 |
| Supr_36 | AAGCCCGTTTCTACATGGTG | TTTGCATACAGTAGCCCTGC | TA | 12 | 104-128 |
| Supr_43 | GAGCGGTGTTGGAAGTTACC | TCAAAGTCCTCTTCATCCGTG | ATG | 2 | 116-119 |
